# Ovarian Biomechanics: From Health to Disease

**DOI:** 10.3389/fonc.2021.744257

**Published:** 2022-01-07

**Authors:** Chenchen Sun, Xiaoxu Yang, Tianxiao Wang, Min Cheng, Yangyang Han

**Affiliations:** ^1^ School of Life Science and Technology, Weifang Medical University, Weifang, China; ^2^ Department of Physiology, Weifang Medical University, Weifang, China

**Keywords:** ovary, biomechanics, signal transduction, ovarian cancer, follicle development

## Abstract

Biomechanics is a physical phenomenon which mainly related with deformation and movement of life forms. As a mechanical signal, it participates in the growth and development of many tissues and organs, including ovary. Mechanical signals not only participate in multiple processes in the ovary but also play a critical role in ovarian growth and normal physiological functions. Additionally, the involvement of mechanical signals has been found in ovarian cancer and other ovarian diseases, prompting us to focus on the roles of mechanical signals in the process of ovarian health to disease. This review mainly discusses the effects and signal transduction of biomechanics (including elastic force, shear force, compressive stress and tensile stress) in ovarian development as a regulatory signal, as well as in the pathological process of normal ovarian diseases and cancer. This review also aims to provide new research ideas for the further research and treatment of ovarian-related diseases.

The mammalian ovary is a major reproductive organ, ensuring various functions essential to the reproductive process. Its most obvious role is the production and release of functional female gametes, oocytes. Growing evidence indicates that the growth and development of the ovary, as well as the malignant process, are accompanied by changes in biomechanical characteristics, but only superficially. Here, we review the roles of biomechanics in normal ovarian development and ovarian diseases, aiming to provide new insights into understanding the mechanisms of ovarian biomechanics.

## Follicle Development and Mechanical Factors

As one of the main reproductive organs of the female reproductive system, the growth and development of the ovary are extremely complex processes involving the participation of multiple regulatory mechanisms, regulatory factors and effect factors. Additionally, some studies have suggestion that mechanical factors are involved in normal ovarian development and the malignant process of ovarian cancer in mammals and play vital roles (1–5). Based on the role of biomechanical factors in the growth and development of various tissues and organs of the human body, their influence on the cell and tissue levels of bone (6), muscle (7), intestine (8), joints (9) and other organs has been studied in depth. Additionally, location shifts and tissue remodeling occur during follicle formation, suggesting that extracellular matrix (ECM) plays an important role in follicle development and that the mechanics involved in the process of ovarian growth and development have gradually attracted increased attention (10, 11).

### Change in the Mechanical Environment During Follicle Development

**Figure 1:**
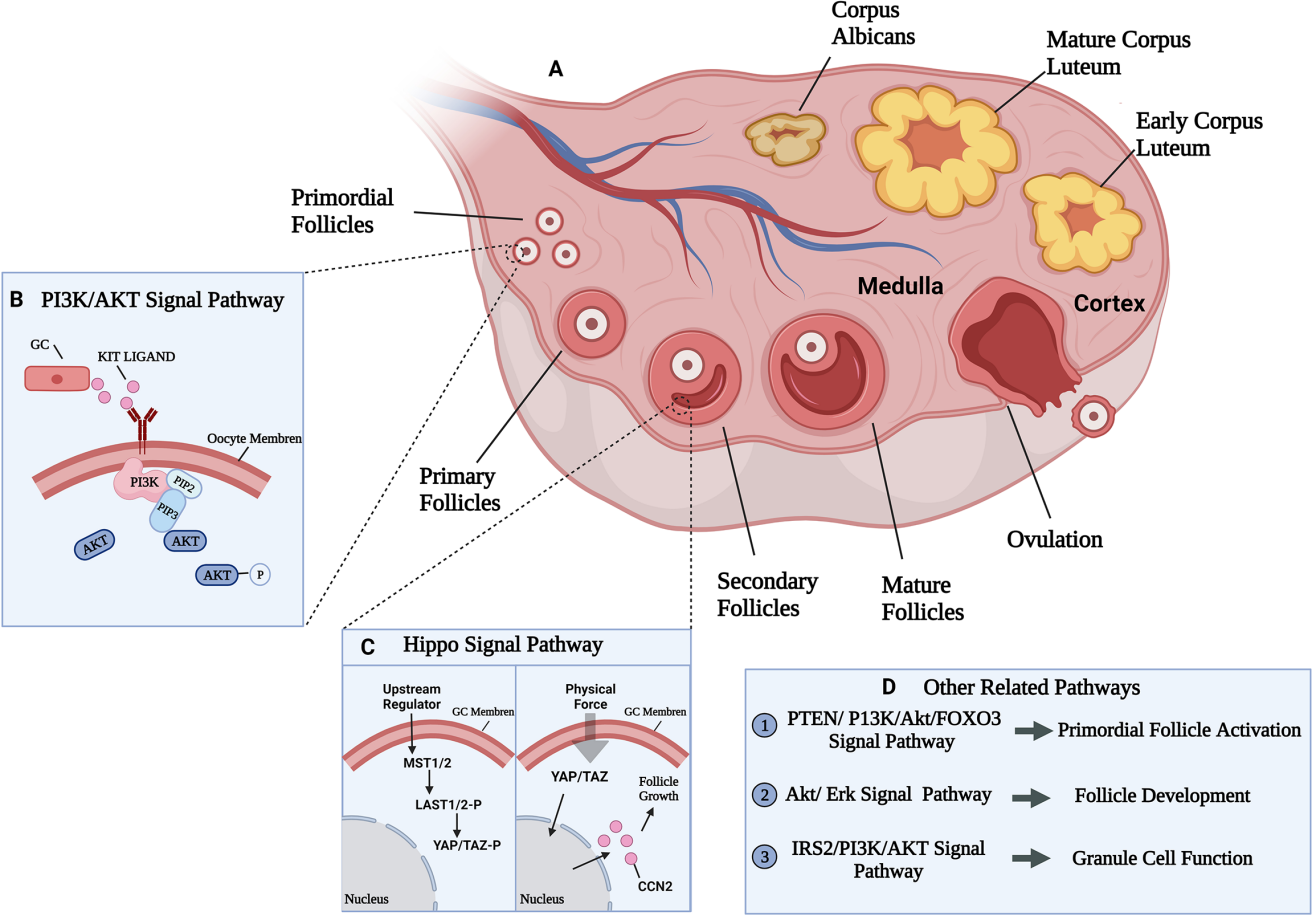
Ovarian developmental processes and some of the mechanical pathways involved. **(A)** The growth and development process of follicle and related signal pathways. Follicle development goes through primordial, primary, secondary, and mature follicles four stages. **(B)** PI3K/AKT signal pathway participate in primordial follicles activation. PI3K was activated by growth factor KIT LIGAND (KL) and converted phosphatidylinositol 4,5-bisphosphate (PIP2) into phosphatidylinositol 3,4,5-trisphosphate (PIP3) at the cell membrane, leading to AKT phosphorylation and activation, eventually lead to primordial follicles activation (14). **(C)** Activated state of Hippo signaling in granulosa cells (left). Uncharacterized upstream factors stimulate the Hippo signaling cascade (MST1/2 and LATS1/2) to phosphorylate and restrict the activition of two transcriptional coactivators (YAP and TAZ). Disruption of Hippo signaling in granulosa cells (right). Physical force disrupt the Hippo signaling cascade by translocating un-phosphorylated YAP/TAZ into the nucleus to bind the TEAD transcriptional factors, thus induce expression of CCN which are involved in granulosa cell proliferation and survival, leading to follicle growth (15). **(D)** Other related signal pathways in follicle development.

The follicle is the fundamental function tissue of mammalian ovary. According to their morphological and functional characteristics, follicles can be divided into primordial, primary, secondary, and mature follicles. Human follicle development begins in the fetal period, from the primal follicle to the primary follicle transition. Primordial follicles are composed by oocytes and pre-granular cells which are single spindle-shaped layer like (12). When stimulated by biological signals, the primordial follicles are recruited into the growth pool, and the shape of granular cells transform from spindle-flat to cubic thus form primary follicles. Later, granular cells proliferate from monolayer to multiple layers with appearance of fluid cavities with different sizes which further synthesize follicular cavities, and at that time, secondary follicles are formed (13).Further, the secondary follicles develop to antral follicles under the regulation of follicle-stimulating hormones (FSH) and finally arrive the ovulatory preparation period (**Figure 1A**). Overall, the follicles development process is mainly comprised the increased number of granulosa cells and the enlargement of oocytes.

In the process of follicles development, some factors were confirmed to involved this. For example, growth factor 9 (GDF 9), bone morphogenetic protein 15 (BMP 15) and BMP 6 were thought to be involved in the recruitment of primordial follicles (16, 17), and GDF 9 and BMP 15 were involved in the regulation of oocyte maturation and the function of granulosa cell (17); Furthermore, Vendola K et al. (18) found that insulin-like growth factor I receptor (IGFR) was increased in oocytes after androgen treatment, which stimulated the activation of primordial follicles and the formation of primary follicles (18).

In all stages of follicle development, mechanical factors are involved, affecting the entire process. Normal ovarian structure is divided into two parts: the cortex and the medulla. Different structural areas provide different hardness environments for primordial follicles: the ovarian cortex comprises connective tissue, which occupies most of the ovary; however, the medulla is located in the center of the ovary and comprises loose connective tissue (19). In general, the rigidity of the cortex is greater than that of the medulla, providing a rigid environment for the complete preservation of primordial follicles. With the production of secondary follicles, the rigid environment of the ovarian cortex limits the maturation and expansion of the follicles, and the secondary follicles then transfer from the cortex to the medulla to obtain a softer environment (20). Additionally, in the process of follicles development, the zona pellucida consisted with the mucopolysaccharide which secreted by granular cells became thickened, and the structure became denser, it suggested that the matrix environment which surround granulosa cells has changed. As early as 1996, a study compared the activation ratio of primordial follicles *in vivo* to primordial follicles *in vitro* in cattle and found that the activation of primordial follicles *in vitro* was significantly better, implying that the ovarian matrix environment plays a particular role in the activation and growth of follicles (21). Felder S et al. (22) used the pore structure formed by a macroporous alginate (Alg) scaffold to simulate the physical environment of the ovarian cortex, regulate the development of follicles, and successfully promote the growth of ovarian primitive follicles *in vitro* (22), suggesting that the internal mechanical structure of the ovary may affect the development of the ovary.

In the process of follicle activation and development, the expression of genes that regulate matrix hardness is in a particular dynamic state. The fibrillin 3 (FBN3) gene regulates the expression of extracellular matrix (ECM) -related proteins and fibrin, uses the transforming growth factor β (TGF-β) signaling pathway to indirectly affect the function of fibroblasts in the matrix, and regulates the production and deposition of collagen (Col) in normal and fibrotic tissues (23). In the early stage of follicle development, during the transition from primordial to primary follicles, FBN3 is expressed in the stromal area around the follicle (24), implying that this gene may regulate the content of collagen to regulate the mechanical environment around the follicle, thereby affecting follicle development.

Not only the above influence factors, some literatures demonstrated that the biomechanics of basement membrane (BM) affected the development of follicles. The BM regulates and maintains the morphology of the epithelium, and its structure changes in different follicular development periods. Chlasta J et al. (25) found that in late follicular development, the follicular morphology changes from cuboidal to squamous or columnar increase the density and growth of follicular fibers then resulting the increased stiffness of BM caused by the embedded of follicular fibers. This change in stiffness creates areas with alternating soft and hard biomechanics which furthermore maintain the follicular morphology (25). Additionally, pre-ovulatory follicles are separated from the ovaries and placed under hydrostatic pressure (HP), and an increase in the number of mature oocytes, indicating that HP promotes oocyte maturation and follicle development (26). The effect of the mechanical environment on the follicle is not limited to the internal environment of the ovary but is in a state where follicle development can be regulated at all times.

### Mechanical Signal Regulation in Follicle Development

The influence of mechanical factors on follicle development is finally realized through the regulation of well-known classical mechanical pathways, such as PI3K or Hippo pathways. Researchers have demonstrated that phosphoinositide 3-kinase (PI3K)signaling pathway stimulation induced the activation of the primordial follicle pool that can lead to rapid depletion of the pool and subfertility (27, 28) (**Figure 1B**). In the ovary, most primitive follicles are in dormant state, while when the follicles transfer from stiffer cortex to softer medulla area, the stimulation of Hippo signaling may slow down the growth of follicles (29). Meanwhile, other studies confirmed that Hippo mechanical signal in the follicles near the cortex may be blocked because of the destruction of the highly rigid microenvironment, resulting in increased Yes-associated protein (YAP) activity and connective tissue (CCN) growth factors secretion, resulting in activation and maturation of primordial follicles and follicle development (15, 30) (**Figure 1C**).

Moreover, studies also confirmed the major regulation of Akt and Erk signaling pathways in follicle growth and development (**Figure 1D**). Ryan K et al. (31) found that inhibition of Akt and Erk pathways inhibit the stimulatory actions of follicle-stimulating hormone (FSH) and insulin-like growth factors (IGF), and thus influence the growth of cultured follicle cells *in vitro* using the inhibitor of Akt or Erk (31). Besides, decreasing the activation of Akt resulted the lower synthesis of estradiol and progesterone and thus slowing down granulosa cell development (32). The combination of ovarian fragmentation and Akt stimulant increases the production of secondary follicles and pre-ovulatory follicles, and trigger actin polymerization and block Hippo signaling (33), it is suggested that Akt and Hippo signaling pathways may interactive in follicles development.

## Mechanical Characteristics of ECM and Follicle Development

The ECM exists in all tissues and organs and comprises mainly water, protein (collagens, elastin, fibronectin, laminins, and several other glycoproteins) and polysaccharides (34). Not only acts as physical supporting role, ECM also has crucial roles because of its special structure. Specifically speaking, various receptors are found on the ECM that mediate different physiological processes. Besides, the ECM of different tissues or organs has a specific structural composition and heterogeneity (35). Studies have showed that in the early stage of stem cell differentiation and early embryonic development, the content of elastin, collagen, BM protein and laminin, increase, providing suitable rigidity for the growth and development (36). Furthermore, similarity function was found in ovary.

### Changes in the Mechanical Characteristics of the ECM During Follicle Development

Changes of ECM elasticity can produce mechanical signals, and such signals not only change the biological characteristic of ECM, but also result in changes of stress fibers and cytoskeleton, which may further regulate the activity level of YAP/TAZ (37). YAP/TAZ is then activated and transferred to nucleus and combines with transcription factors to regulate gene expression (38, 39). Other research found that stiffer rigidity of ECM activates TGF-β, and this activation is mediated by the high contractility produced by a-ama-positive stress fibers *via* integrins to the large latent complex (LLC), which releases TGF-β1 when anchored in the stiff ECM. This activation acts to induce myofibroblasts (Mfs) differentiation, which then regulate normal physiological processes of cells or pathological fibrosis, but with reduced activation of TGF-β1 compared to softer matrices (40, 41).

The preservation of oocytes depends on the ovarian environment. Oocytes are in dormant state in the ovary, and the surrounding granular cells and ECM are the main sources of mechanical stress on oocytes. Specificity speaking, the abundant of actin filaments in granular cells produce pressure on oocytes to keep them in dormant state, and the fibronectin and collagen type IV in cortical ECM region which around the perifollicular area also maintain the dormant state. Nagamatsu G et al. (42) found that the number of activated oocytes increased after using collagenase type IV and trypsin to elimination collagen type IV and fibronectin, and the number of large oocytes (>50 μm in diameter) also increased under similar condition (42), demonstrated the crucial role of ECM in maintain oocytes’ dormant state.

Following the activation of follicle development, the mechanical microenvironment of ECM appears to be different. Human prepubertal ECM is stiffer caused by the higher percentage of thick collagen fibers in prepubertal ovary, and further reason is the decreased elasticity of thick collagen fibers make ECM less compliant and more rigid (20). Combining the mechanical characteristics of follicle development, the stiffer prepubertal ECM environment may provide suitable environment for preservation of primordial follicles.

Excepting the collagen, ECM also has other components, such as hyaluronic acid (HA) and elastin, while their changes will also lead to the mechanical changes of ECM. HA is a glycosaminoglycan, and as the main component of the ECM, it is widely confirmed that the changes of HA will alter the mechanical properties of ECM (43–45). Besides, HA also plays a certain role in cell differentiation and cell growth (46), promoting muscle cell proliferation and other physiological functions (47). During follicle development, high concentrations of low-molecular-weight hyaluronic acid can cause abnormal meiotic abnormalities and abnormal aging of oocytes (48), and it may also due to the mechanical changes of ECM. As another major component of ECM, the content of elastin also changes in different stages of ovarian development. The synthesis of elastin in the adult ovary gradually stops, and the content is lower than that in the prepubertal ovarian elastin (20). Considering the important role of elastin in mechanical homeostasis (49, 50), the changes of elastin synthesis may alter the mechanical properties of ECM and then play a role in ovarian development.

### ECM Simulation of Ovarian Development *In Vitro*

In order to research the influence of ECM on ovarian development, many studies have attempted to use biological materials with different properties to create a suitable physical environment for ovarian development to achieve ovarian culture *in vitro*. For example, researchers commonly used biological materials, such as hyaluronic acid and Alg, form a matrix environment with different stiffnesses to support follicle development. Kim et al. (51) constructed a 3D culture of ovarian follicles and found that Alg which has harder rigidity limiting follicle development compared with the hydrogel formed by hyaluronic acid which has softer rigidity (51). He et al. (52) produced a new ECM materials composed of different concentration collagen and Alg, and found that compared with the softer ECM composition (0.5% collagen core and 2% Alg shell), ovarian tissue cultured on stiffer ECM composition (0.5% Col plus 0.5% Alg as the core and 2% oxidized alginate shell) produced at least 10% higher estradiol, and the development rate of bionic ovarian micro-tissues was also increased (52), indicating the mechanical characteristic of ECM played a major role in ovarian development.

Furthermore, the mechanical microvibration culture (MVC) system was used to culture human immature oocytes *in vitro* and successfully obtained normal mature oocytes with development potential comparable to mature oocytes (53). During the construction of the above ovarian *in vitro* culture ECM model, the development of the ovary was simulated by changing the ratio of the construction materials, showing that different ECM structures have different effects on the developmental rate of the ovary. Confirm the importance of the ECM mechanical environment in the normal development of the ovary, and to further understand the role of the ECM in ovarian development, indicating that it is necessary to further explore the specific mechanism by which the ECM mechanical environment regulates ovarian development.

## Ovarian Cancer and Biomechanics

As a global problem, ovarian cancer is no longer regarded as a single disease but as a heterogeneous disease with different histological subtypes and different identifiable risk factors, origin cells, molecular and clinical features and treatments (54). Among them, epithelial ovarian cancer has the highest incidence, accounting for approximately 90% of ovarian cancer (55). Ovarian cancer is usually diagnosed at an advanced stage because of the lack of an effective early screening strategy. However, with the continuous research and progress of genetic testing based on personal or family history, ethnic background or other demographic characteristics, some individuals with a high risk of ovarian cancer, such as those with germline mutations in BRCA1 or BRCA2, have been identified. However, we did not see any improvements in mortality rates (56). Presently, ovarian cancer treatment is dominated by surgery and platinum chemotherapy. Angiogenesis inhibitors, poly (ADP-ribose) polymerase (PARP) inhibitors and immunotherapy (cytotoxic T lymphocyte antigen 4 and PD-1) have updated the treatment model of ovarian cancer to a certain extent, while most of them are still in preclinical trials (57). Effective methods of prevention and early screening as well as promising new treatment models remain to be explored, and the study of biomechanics has shown potential in this field.

### Elastic Changes of Ovarian Cancer Cells

Studies have shown that ovarian cancer cells are different from normal cells and healthy cells in growth, adhesion, morphology and cytoskeleton structure (58, 59). This finding is partly due to cell biomechanics (elastic and viscous behavior), the study of which is crucial to the development of disease treatments and detection methods. Cell biomechanics is an important part of biology. It involves the study of cell membrane, cytoskeleton deformation, elastic constant, viscoelasticity, adhesion and other mechanical properties under mechanical load, and the effects of mechanical factors on cell growth, development, maturation, proliferation, senescence and death and its mechanism. It is the basis and key point to reveal the life activities of cells. As more physical properties of cells are investigated, such as growth, adhesion, morphology and cytoskeleton structure we mentioned above are all physical properties, different properties may become potentially powerful tools for cancer diagnosis and treatment. Many research groups have calculated the elastic modulus of cancer cells and believe that cancer cells are “softer” or deform faster than healthy, untransformed cancer cells (60, 61). Ovarian cancer is no exception. Although the characterization of information concerning the mechanical properties of human malignant and benign ovarian cells trails that of other cancers because of the lack of advanced detection methods, with the continuous progress of technology, such as ultrasonic elastography and magnetic resonance elastography, a series of studies have also shown a significant relationship between the viscoelasticity and biological properties of ovarian cancer cells.

Ketene et al. (62) relied on the spontaneous transformation of epithelial cells on the surface of mouse ovaries in cell culture and established a primary cell model of progressive ovarian cancer in C57BL/6 mice (62). The results showed an obvious transfer pattern in the early, middle and late transformation of the cell line. When the same cell line changes from the benign to invasive stage, an increasing number of hypoelastic cells are found in the cell population. This high invasiveness, high mobility, low elasticity and viscosity may be related to significant differences in cytoskeletal structure (63).

Both the cell changes in different periods and the properties of different pathological types of ovarian cancer cells have been quantified. The elastic modulus of mucinous ovarian cancer, serous ovarian cancer, mature teratoma and endometriosis was quantitatively classified by atomic force microscopy. Ansardamavandi et al. (64) mechanically characterized human ovarian tissues with four distinct pathological conditions: mucinous, serous, and mature teratoma tumors, and non-tumorous endometriosis by using atomic force microscopy. Mechanical elasticity profiles were quantified and the resultant data were categorized using K-means clustering method, as well as fuzzy C-means, to evaluate elastic moduli of cellular and non-cellular parts of diseased tissues and compare them among four disease conditions. Samples were stained by hematoxylin-eosin staining to further study the content of different locations of tissues. They found that pathological state vastly influenced the mechanical properties of the ovarian tissues. Significant alterations among elastic moduli of both cellular and non-cellular parts were observed. Mature teratoma tumors commonly composed of multiple cell types and heterogeneous ECM structure showed the widest range of elasticity profile and the stiffest average elastic modulus of 14 kPa. Samples of serous tumors were the softest tissues with elastic modulus of only 400 Pa for the cellular part and 5 kPa for the ECM. Tissues of other two diseases were closer in mechanical properties as mucinous tumors were insignificantly stiffer than endometriosis in cellular part, 1300 Pa compared to 1000 Pa, with the ECM average elastic modulus of 8 kPa for both. In short, The higher incidence of carcinoma out of teratoma and serous tumors may be related to the intense alteration of mechanical features of the cellular and ECM, serving as a potential risk factor which necessitates further investigation (64). A similar outcome was found in a study by Chen et al. (65) the elasticity and viscosity of ovarian cancer cells OVCAR-3 (1195.72 ± 122.94Pa) and HO-8910 (996.27 ± 52.56Pa) were significantly lower than those of human ovarian surface epithelial cells (2160.94 ± 167.77 Pa) (65). Further detection showed that the migration/invasion ability of OVCAR-3 and HO-8910 cells was significantly higher than that of the control group.

**Figure 2:**
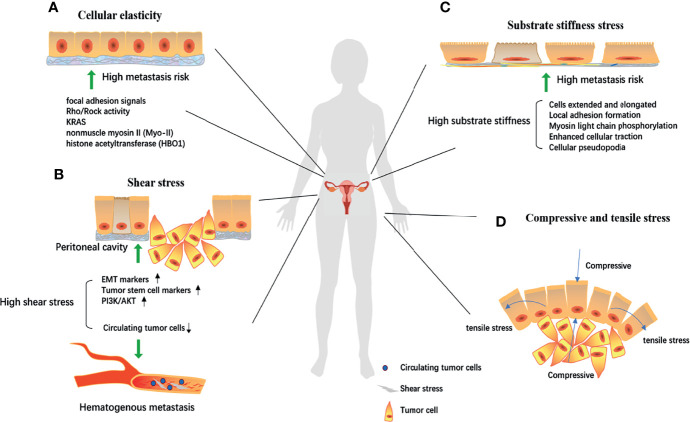
The biomechanical mechanism of ovarian cell canceration and metastasis. **(A)** Focal adhesion signals, Rho/Rock activity, KRAS, nonmuscle myosin II (Myo-II), histone acetyltransferase (HBO1) could reduce cell elasticity through signal transduction, thereby increasing the risk of metastasis; Under high shear environment, the epithelial-mesenchymal transformation and tumor stem cell markers are significantly expressed. **(B, C)** The abnormal activation of the PI3K/Akt pathway significantly increases the malignancy of cells. While in hematogenous metastasis, high shear stress destroyed circulating tumor cells and prevented cancer metastasis; Local adhesion formation, myosin light chain phosphorylation, enhanced cell traction, and cellular pseudopodia produced by extracellular matrix tissue increase substrate stiffness, gradually stretch and elong the cells, thereby making ovarian cancer cells have a high risk of metastasis. **(D)** Proliferation of ovarian cancer cells will produce tension stress and compressive stress on the cells around the developing mass and the increase of these two stress will further increase the invasion ability of tumor cells. EMT, epithelial-mesenchymal transformation.

Based on the effective phenotypic changes, that is, the physical structure is sufficient to affect the behavior of cancer cells, exploration of the mechanism of “low cellular elasticity-high metastasis risk” was also gradually conducted. Differences in focal adhesion signals (higher FAK expression) and Rho/ROCK activity (increased RhoA activity) suggest that they are involved in biomechanically driven cellular responses (66), KRAS (67), nonmuscle myosin II (Myo-II) (68), and histone acetyltransferase (HBO1) (69) have also been studied to change the mechanical phenotype of ovarian cancer cells through signal transduction (**Figure 2**), and the change of cellular elasticity was measured directly by atomic force microscopy. Thereby changing the metastatic tendency of ovarian cancer. According to this physiological characteristic, the discovery and application of selenium nanoparticles (70) and gold nanoparticles (71), which can change the mechanical characteristics of the cell membrane, may provide a new idea for ovarian cancer treatment.

### Ovarian Cancer and Shear Stress

Whether because of the continuous peristalsis of the gastrointestinal system or exposure to the ascites environment, the surface of the ovary is exposed to peritoneal pressure and shear force, creating a mechanical microenvironment for the ovarian cancer cells (72). The study of the effect of shear stress on the occurrence and development of ovarian cancer is accompanied by the progress of corresponding experiments and mathematical models; particularly, there is a need for computer simulation models to improve our understanding of the physiological stress that occurs in the peritoneal cavity. Because the movement of the body is directly related to the level of shear stress experienced by floating and attached ovarian cancer, these complex interactions must be modeled and analyzed using hydrodynamic systems (73).

The *in vitro* progression model of ovarian cancer is widely used in the study of shear stress. Hyler et al. (74) conducted experiments to test the different metastatic potentials of mouse ovarian cancer cell lines under the influence of low-level shear stress (74). Benign to highly invasive epithelial cell lines of ovarian cancer were used. OCE1 (healthy person) and SKOV3 (human ovarian clear cell adenocarcinoma) cells were exposed to fluid shear stress < 1 dyne/cm2 on a rotating plate for 12 days. Even with low-intensity shear, genomic changes were observed in the treated cells. Shear stress may not only be a contributing factor to the progression of ovarian cancer but also a key factor in benign cell deterioration, which leads to changes in the mechanical phenotype of cells and is associated with the risk of ovarian cancer metastasis (**Figure 2**). Specifically, after exposure to three consecutive 96 h (288 h total) fluid shear stress, a significant increase in CREST-positive (chromosome mis-segregation) micronuclei was observed in all cell lines (p < 0.001), as well as low levels of CREST-negative (DNA fragments) micronuclei observed in SKOV-3 (0.4% ± 0.1%) cells. Besides, in non-tumorigenic MOSE-E cells, fluid shear stress may promote the generation of tetraploid cells by inducing defective cell division. Researchers also studied spheroid formation, which reflects a more metastatic phenotype, for it was related to change of cytoskeleton organization. The tumorigenic cells lines (SKOV-3) responded to fluid shear stress by detaching and forming aggregates or spheroids. SKOV-3 spheroids grew in diameter from 139.5 ± 10.6μm to 208.6 ± 87.8μm after repeated passaging and were able to re-attach to the culture dishes with a moderate adherent monolayer outgrowth. A model that mimics peritoneal fluid pressure and shear stress caused by continuous peristalsis of the gastrointestinal system was constructed by Avraham-Chakim et al. (75) based on fluid-induced wall shear stress exposure in epithelial ovarian cancer (75). The authors used OVCAR-3 cell line, cultured them in a simulated physiological environment, applied wall shear forces of 0.5, 1.0, and 1.5 dyne/cm2 on the cell surface, and then studied the changes in mechanical indexes, such as the cytoskeleton and actin. Formation of a thick filamentous network of stress fibers was observed in cells exposed to shear stress, compared with control cultures in which actin was present mainly in the peripheral areas of the cell. There was a linear relationship between the magnitude of wall shear stresses and the level of stress fibers formation and that the level of stress fibers formation depended on the magnitude of shear stress. Furthermore, the formation of stress fibers also leads to cell elongation. These conditions occur during the occurrence and development of tumors. For example, a similar outcome of shear stress induced actin-tubulin remodeling was also observed in metastatic esophageal cancer cells (76). It suggests that stress fibers may be associated with the development of ovarian cancer.

The development and metastasis of ovarian cancer caused by increased shear stress must be accompanied by potential cellular and molecular mechanisms. Researchers have noticed this and performed a series of studies on the related mechanisms. Ip CK et al. (77) found that epithelial-mesenchymal transformation and expression of tumor stem cell markers can be obtained when ovarian cancer cells grow under fluid shear stress, indicating a significant increase in the degree of malignancy of ovarian cancer cells (77). The same results were also observed in Rizvi’s et al. study (78). Additionally, these cells developed significant chemotherapy resistance to cisplatin and paclitaxel, a finding that may be attributed to abnormal activation of the PI3K/Akt pathway.

However, during process of hematogenous metastasis, the opposite results was found. High shear stress destroyed circulating tumor cells and prevented cancer metastasis. Studies have shown that a high shear stress of 60 dyne/cm2 during intensive exercise can kill more than 90% of circulating tumor cells within 4 hours. Compared with the low shear stress of 15 dyne/cm2 at rest, long-term high shear stress treatment effectively reduces the survival rate of highly metastatic and drug-resistant cancer cells (79). The killing effect of this high shear stress on cancer cells can be blocked by platelets (80). Specifically, when platelets are under shear stress, lactate dehydrogenase is significantly decreased, indicating that the nondestructive cycle time of cancer cells is prolonged *in vivo*.

The reverse effect of this shear stress in the process of *in vitro* and hematogenous metastasis is an interesting and contradictory discovery, suggesting the dual mechanism of shear stress in a complex body environment: on the one hand, continuous shear stress can cause changes in the cell genome and mechanical phenotype, leading to the deterioration of normal cells and metastasis of cancer cells; on the other hand, high shear stress can destroy cancer cells in the circulatory system and reduce the risk of cancer metastasis. Future research should be more targeted, specifically describing the mechanism of shear stress under different environmental conditions.

### Ovarian Cancer and Substrate Stiffness Stress

The ECM provides physical support for tissues, organs and signaling pathways through integrins and other membrane receptors (81). This characteristic also determines its key position during tumor metastasis. A series of studies have been performed to understand the interaction between the occurrence and development of cancer cells and the physical properties of the substrate (76–80).

Cells are very sensitive to substrate stiffness and texture. To explore the cell response to these two types of input in a precisely controlled way, Park et al. (82) analyzed the substrate response of Chinese hamster ovary cells to different stiffnesses (from 1.8 MPa to 1.1 GPa). With increasing substrate stiffness, the cells gradually extended and elongated in a dependent manner (82). This change was partly attributed to local adhesion formation, myosin light chain phosphorylation and enhanced cellular traction (83), as well as cellular pseudopodia produced by ECM tissue under strong substrate stiffness (84), which is a prelude to metastasis. The increase in substrate stiffness may be closely related to the distant metastasis of ovarian cancer cells (**Figure 2**). The epithelial-mesenchymal transformation pathway (85) and the Rho/ROCK pathway (86) may play important regulatory roles.

### Ovarian Cancer and Compressive and Tensile Stress

Ovarian cancer cells are subjected to a series of compression and stretch stimuli that contribute to mechanical transduction. On the one hand, as ovarian cancer expands, it produces tension on the cells around the developing mass; on the other hand, the cells are exposed to chronic loads from abdominal cavity, surrounding cell displacement and growth-induced stress (87). We can understand that when the body surface is swollen, inflammatory exudation or congestion will produce a squeezing force on the skin, and the skin will also have a counteracting squeezing force on the inside, which is compressive stress. Meanwhile, the normal surface of the skin expands and the muscle fibers pull each other, resulting in tensile stress (**Figure 2**). Relatively few studies have investigated the tensile stress of ovarian-specific cells (88). The regulation of the RhoA/ROCK signaling cascade seems to be critical and further plays an important role in vascular endothelial growth factor-mediated angiogenesis (89).

Studies on the effect of compressive stress on cancer progression have found a trend toward late invasion and metastasis phenotypes. Klymenko et al. (87) used an *in vitro* model system to simulate the effects of static pressure on four ovarian cell lines under ascites pressure for 6 or 24 hours. The enhancement of invasive ability was accompanied by an increase pressure, and the expression of genes related to epithelial-mesenchymal transformation and dispersion of multicellular aggregates was regulated by compressive stress to varying degrees. It was noteworthy that the expression of Epithelial (E)3-cadherin (e-cadherin) and neural (N)-cadherin (n-cadherin) were changed under static pressure, further affect cell-cell and cell-matrix adhesion, while no changes of cell proliferation related signaling pathways were observed (87). Burkhalter et al. (88) proposed that integrin induced e-cadherin internalization and activated β-catenin, thus co-regulated intercellular linkage mainly according to Wnt/β-catenin signaling pathway in ovarian cancer invasion and metastasis. And further investigate indicated that these influences might cause by the changed expression of membrane type 1 matrix metalloproteinases (MMP-14) (88). In general, the response of Wnt signaling pathway under compressive and tensile stress may be one of the potential molecular mechanisms in ovarian cancer invasion and metastasis. In a similar study, Novak et al. (90) encapsulated high-grade serous ovarian cancer cell lines in simulated ECM hydrogels containing agarose collagen and type I collagen, and used a 3D compression bioreactor to observe the effects of compressive stress on ovarian cancer cells under limited cyclic or static pressure for 24 and 72 hours, respectively. Compression stimulation not only significantly increased the proliferation and invasion of ovarian cancer cells but also caused chemotherapy resistance in ovarian cancer cells, which may be attributed to the abnormal activation of CDC42 (90). Notably, whether the stem cell characteristics of ovarian cancer cells will appear under compressive stress and promote development and metastasis warrants further study.

### Biological Characteristics of Ovarian Cancer Spheroids

Multicellular spheroids (MCS) also need to be mentioned. Ovarian cancer cells typically spread and metastasize within the abdominal cavity, where ascites promotes this process. Metastatic ascites contains MCS, which reduces the sensitivity of the body to chemotherapy (91). Through *in vivo* lineage tracing, the researchers found that MCS appeared preferentially from collective detachment, rather than aggregation in the abdomen (92). MCS usually appears as “blastuloid” spheroids after maturation, with a smooth profile and immotile cells (93). It is a dynamic structure composed of proliferating, non-proliferating and hypoxic regions. The spheroid neither binds nor allows cells to penetrate (94). This form can keep the internal cells from being damaged by external forces such as shear stress, and maintain phenotypic heterogeneity during dissemination (92).

## Benign Ovarian Diseases and Biomechanics

### Polycystic Ovary Syndrome

Polycystic ovary syndrome (PCOS) is a common endocrine disorder with many pathological manifestations, such as hormone disorders, insulin resistance, and ovarian dysfunction (oligoovulation, anovulation or polycystic ovary). Among them, insulin resistance may cause infertility (95); when the two pathological characteristics of hyperandrogenism and ovarian dysfunction are met, it can be diagnosed as PCOS (24).

With the restoration of Hippo signal transduction in the ovary, the growth of follicles slows down. Therefore, CCN growth factor treatment of these patients or topical application of actin polymerization drugs may offset the impact of Hippo on the ovaries and provide potential treatments for patients with PCOS. Additionally, the high androgenic properties of PCOS may be caused by a rigid biomechanical environment. In a mouse model, androgens secreted by follicles in a harder and denser Alg substrate were present at higher levels (96).

The dense collagenous and thickened ovarian cortex may create a biomechanically forbidden environment and change the mechanical signals in the ovary (29). Forbidden environment means the stiff cortical region, which could decrease local Hippo signaling, leading to overactivity of YAP, increased CCN growth factor secretion, stromal cell proliferation, and thecal cell hyperplasia. The follicles of patients with PCOS have polycystic ovaries with an increase in matrix hypertrophy; the granular cell substrate is thickened, and the electron density in the zona pellucida formed by secondary follicles increases. Ovarian drilling is often used for clinical treatment, and the mechanical forces generated in the process cause changes in the ovarian endocrine level, especially an luteinizing hormone decline (97, 98). Presently, a mechanobiology-based treatment that ruptures the ovarian cortex through *in vitro* activation (IVA) temporarily interrupts the Hippo pathway and promotes the secondary development of follicles. Compared with traditional surgical treatment, this mechanical biological therapy reduces damage to the ovaries (15).

### Ovarian Insufficiency

Ovarian insufficiency is a key fertility defect characterized by impaired expected follicular reserve (99). This defect occurs when the existing primordial follicles are depleted or inhibited in activation (100), and its pathophysiological mechanism has not yet been determined. Studies have shown that by obtaining primordial follicles in the ovarian cortex, autologous transplantation after the activation of mechanical destruction *in vitro* can stimulate their development into preantral follicles (101). Additionally, the interaction between the Akt pathway and mechanical signals is crucial for follicle activation, and this understanding has been applied to some treatments that may maintain female fertility.

## Conclusion

As a complex and relatively stable system, the development of ovaries and follicles has a profound impact on female fertility and human reproductive potential, and its entire biological behavior is mediated by the participation of biomechanics. Follicle maturation, ovarian development, and the occurrence and development of ovarian cancer all involve extensive interventions of mechanical signals. The elucidation of biomechanics is critical for enhancing our understanding of the pathophysiology of ovaries and follicles. Future research is expected to further characterize biomechanics and related signal transduction pathways.

## Author Contributions

CS and XY wrote most of the manuscript. TW wrote parts of the manuscript and revised the manuscript. MC provided all supporting for this review. YH provided the concept and organized members to discuss the manuscript. All authors contributed to the article and approved the submitted version.

## Funding

The present study was supported by the National Natural Science Foundation of China (grant numbers: 81501683) and the Natural Science Foundation of Shandong Province (grant No. ZR2019MH047, ZR2015HL057).

## Conflict of Interest

The authors declare that the research was conducted in the absence of any commercial or financial relationships that could be construed as a potential conflict of interest.

## Publisher’s Note

All claims expressed in this article are solely those of the authors and do not necessarily represent those of their affiliated organizations, or those of the publisher, the editors and the reviewers. Any product that may be evaluated in this article, or claim that may be made by its manufacturer, is not guaranteed or endorsed by the publisher.

## Abbreviations

IGFR, insulin-like growth factor I receptor; FBN3, fibrillin 3; GDF 9, factor 9; BMP 15, bone morphogenetic protein 15; TGF, transforming growth factor; BM, basement membrane; HP, hydrostatic pressure; PI3K, phosphoinositide 3-kinase; YAP, Yes-associated protein; CCN, connective tissue growth factors; FSH, Follicle-stimulating hormone; IGF, insulin-like growth factors; PDMS, polydimethylsiloxane; DSL, Delta, Srrate, Lag2; LLC, large latent complex; Mfs, myofi broblasts; HA, hyaluronic acid; GAGs, glycosaminoglycans; 3D, three-dimensional; AKT, protein kinase B; RPS6, ribosomal protein S6; ERK, extracellular regulated protein kinases; SHP2, Src-homology-2-containing phosphotyrosine phosphatase; ECM , Extracellular matrix; Col, collagen; Alg, alginate; MVC, microvibration culture; PARP, poly (ADP-ribose) polymerase; Myo-II, nonmuscle myosin II; HBO1, histone acetyltransferase; PCOS, Polycystic ovary syndrome; IVA, in vitro activation; e-cadherin, Epithelial (E)3-cadherin; n-cadherin, Neural (N)-cadherin; MMP-14, Membrane type 1 matrix metalloprotein.
